# Supplementary material for: Multi-scale V-net architecture with deep feature CRF layers for brain extraction
Source: Commun Med (Lond). 2024 Feb 23;4:29. doi: 10.1038/s43856-024-00452-8 (PMC10891085; doi:10.1038/s43856-024-00452-8)
Supplement: Supplementary file 2 — Reporting Summary [file 43856_2024_452_MOESM2_ESM.pdf]

nature portfolio

Corresponding author(s): Jong Sung Park

Last updated by author(s): Oct 30, 2023

Reporting Summary

Nature Portfolio wishes to improve the reproducibility of the work that we publish. This form provides structure for consistency and transparency in reporting. For further information on Nature Portfolio policies, see our Editorial Policies and the Editorial Policy Checklist.

Statistics

For all statistical analyses, confirm that the following items are present in the figure legend, table legend, main text, or Methods section.

n/a	Confirmed	
☒	☐	The exact sample size (*n*) for each experimental group/condition, given as a discrete number and unit of measurement
☐	☒	A statement on whether measurements were taken from distinct samples or whether the same sample was measured repeatedly
☒	☐	The statistical test(s) used AND whether they are one- or two-sided<br>*Only common tests should be described solely by name; describe more complex techniques in the Methods section.*
☒	☐	A description of all covariates tested
☒	☐	A description of any assumptions or corrections, such as tests of normality and adjustment for multiple comparisons
☐	☒	A full description of the statistical parameters including central tendency (e.g. means) or other basic estimates (e.g. regression coefficient) AND variation (e.g. standard deviation) or associated estimates of uncertainty (e.g. confidence intervals)
☒	☐	For null hypothesis testing, the test statistic (e.g. *F*, *t*, *r*) with confidence intervals, effect sizes, degrees of freedom and *P* value noted<br>*Give P values as exact values whenever suitable.*
☒	☐	For Bayesian analysis, information on the choice of priors and Markov chain Monte Carlo settings
☒	☐	For hierarchical and complex designs, identification of the appropriate level for tests and full reporting of outcomes
☒	☐	Estimates of effect sizes (e.g. Cohen's *d*, Pearson's *r*), indicating how they were calculated

Our web collection on statistics for biologists contains articles on many of the points above.

Software and code

Policy information about availability of computer code

Data collection	We did not collect any datasets. We used public datasets. See section Data.
Data analysis	Dice coefficient and Jaccard index were calculated according to the exact equation using python's numpy 1.22.4 package. Hausdorff distance was calculated using scikit-image 0.18.3 package.

For manuscripts utilizing custom algorithms or software that are central to the research but not yet described in published literature, software must be made available to editors and reviewers. We strongly encourage code deposition in a community repository (e.g. GitHub). See the Nature Portfolio guidelines for submitting code & software for further information.

Data

Policy information about availability of data

All manuscripts must include a data availability statement. This statement should provide the following information, where applicable:

- Accession codes, unique identifiers, or web links for publicly available datasets
- A description of any restrictions on data availability
- For clinical datasets or third party data, please ensure that the statement adheres to our policy

All data used in the article are publicly available. CC359: https://www.ccdataset.com/download, NFBS: http://preprocessed-connectomes-project.org/NFB_skullstripped/, HCP: https://www.humanconnectome.org/study/hcp-young-adult/data-releases, FCP-INDI/HBN: http://fcon_1000.projects.nitrc.org/indi/s3/

index.html, OASIS3: https://oasis-brains.org/, IXI: https://brain-development.org/ixi-dataset/, Hammers: https://brain-development.org/brain-atlases/, LPBA40: https://www.loni.usc.edu/research/atlas_downloads

Human research participants

Policy information about studies involving human research participants and Sex and Gender in Research.

Reporting on sex and gender	Not relevant
Population characteristics	Analyzed data include Alzheimers, Lesions and healthy adult data, along with pediatric data. The complete description of the datasets and population characteristics is provided in the links of the Data section.
Recruitment	No participants were recruited. All datasests were publicly available.
Ethics oversight	Each public dataset used has received ethics approvals from the relevant organizations. For example, OASIS 3 is both board certified and HIPAA compliant as reported in https://www.medrxiv.org/content/10.1101/2019.12.13.19014902v1.full.pdf

Note that full information on the approval of the study protocol must also be provided in the manuscript.

Field-specific reporting

Please select the one below that is the best fit for your research. If you are not sure, read the appropriate sections before making your selection.

☒ Life sciences ☐ Behavioural & social sciences ☐ Ecological, evolutionary & environmental sciences

For a reference copy of the document with all sections, see nature.com/documents/nr-reporting-summary-flat.pdf

Life sciences study design

All studies must disclose on these points even when the disclosure is negative.

Sample size	Not applicable. This is not a study design paper.
Data exclusions	Not applicable.
Replication	Not applicable
Randomization	Not applicable.
Blinding	Not applicable.

Reporting for specific materials, systems and methods

We require information from authors about some types of materials, experimental systems and methods used in many studies. Here, indicate whether each material, system or method listed is relevant to your study. If you are not sure if a list item applies to your research, read the appropriate section before selecting a response.

Materials & experimental systems

n/a	Involved in the study	
☒	☐	Antibodies
☒	☐	Eukaryotic cell lines
☒	☐	Palaeontology and archaeology
☒	☐	Animals and other organisms
☒	☐	Clinical data
☒	☐	Dual use research of concern

Methods

n/a	Involved in the study	
☒	☐	ChIP-seq
☒	☐	Flow cytometry
☐	☒	MRI-based neuroimaging

Magnetic resonance imaging

Experimental design

Design type	N/A

Design specifications	N/A
Behavioral performance measures	N/A

Acquisition

Imaging type(s)	structural MRI
Field strength	Depends on the public dataset used. Expected to be in the commonly used 1.5T to 3T range.
Sequence & imaging parameters	Publicly available T1 structural MRI images were used. See links in Data section.
Area of acquisition	The publicly available datasets were acquired in different sites.

Diffusion MRI ☐ Used ☒ Not used

Preprocessing

Preprocessing software	No further preprocessing was performed beyond what the public datasets already provided.
Normalization	Not normalized. The datasets were not registered to a template. We processed in their native space.
Normalization template	Not used.
Noise and artifact removal	Not used.
Volume censoring	Not used.

Statistical modeling & inference

Model type and settings	Not applicable
Effect(s) tested	Not applicable

Specify type of analysis: ☒ Whole brain ☐ ROI-based ☐ Both

Statistic type for inference (See Eklund et al. 2016)	Not applicable
Correction	Not applicable

Models & analysis

n/a	Involved in the study
☒	☐ Functional and/or effective connectivity
☒	☐ Graph analysis
☒	☐ Multivariate modeling or predictive analysis
